# Comparison of four models predicting the malignancy of pulmonary nodules: A single-center study of Korean adults

**DOI:** 10.1371/journal.pone.0201242

**Published:** 2018-07-31

**Authors:** Bumhee Yang, Byung Woo Jhun, Sun Hye Shin, Byeong-Ho Jeong, Sang-Won Um, Jae Il Zo, Ho Yun Lee, Insoek Sohn, Hojoong Kim, O. Jung Kwon, Kyungjong Lee

**Affiliations:** 1 Division of Pulmonary and Critical Care Medicine, Department of Medicine, Samsung Medical Center, Sungkyunkwan University School of Medicine, Seoul, South Korea; 2 Department of Thoracic and Cardiovascular Surgery, Samsung Medical Center, Sungkyunkwan University School of Medicine, Seoul, Korea; 3 Department of Radiology, Samsung Medical Center, Sungkyunkwan University School of Medicine, Seoul, Korea; 4 Statistics and Data Center, Samsung Medical Center, Seoul, Korea; University of Manitoba, CANADA

## Abstract

**Objective:**

Four commonly used clinical models for predicting the probability of malignancy in pulmonary nodules were compared. While three of the models (Mayo Clinic, Veterans Association [VA], and Brock University) are based on clinical and computed tomography (CT) characteristics, one model (Herder) additionally includes the ^18^F-fluorodeoxyglucose (FDG) uptake value among the positron emission tomography (PET) characteristics. This study aimed to compare the predictive power of these four models in the context of a population drawn from a single center in an endemic area for tuberculosis in Korea.

**Methods:**

A retrospective analysis of 242 pathologically confirmed nodules (4–30 mm in diameter) in 242 patients from January 2015 to December 2015 was performed. The area under the receiver operating characteristic curve (AUC) was used to assess the predictive performance with respect to malignancy.

**Results:**

Of 242 nodules, 187 (77.2%) were malignant and 55 (22.8%) were benign, with tuberculosis granuloma being the most common type of benign nodule (23/55). PET was performed for 227 nodules (93.8%). The Mayo, VA, and Brock models showed similar predictive performance for malignant nodules (AUC: 0.6145, 0.6042 and 0.6820, respectively). The performance of the Herder model (AUC: 0.5567) was not significantly different from that of the Mayo (vs. Herder, p *=* 0.576) or VA models (vs. Herder, p *=* 0.999), and there were no differences among the three models in determining the probability of malignancy of pulmonary nodules. However, compared with the Brock model, the Herder model showed a significantly lower ability to predict malignancy (adjusted p *=* 0.0132).

**Conclusions:**

In our study, the Herder model including the ^18^FDG uptake value did not perform better than the other models in predicting malignant nodules, suggesting the limited utility of adding PET/CT data to models predicting malignancy in populations within endemic areas for benign inflammatory nodules, such as tuberculosis.

## Introduction

Pulmonary nodules are being detected with increasing frequency because of the increased use of chest computed tomography (CT) [1,2]. Recent low-dose chest CT screening trials showed a beneficial effect on survival for individuals at increased risk of lung cancer [3–6]. However, the management of pulmonary nodules incidentally detected on CT is a pressing clinical concern because accurately predicting malignant nodules is not straightforward.

Recently, several prediction models using clinical and radiological values have been developed that can help physicians to distinguish between benign and malignant nodules [7]. The classical prediction models (Mayo Clinic [8], Veterans Association (VA) [9], and Brock University [10]) only include clinical values and radiological characteristics on CT, while a fourth model proposed by Herder et al. [11] additionally includes the ^18^F-fluorodeoxyglucose (FDG) uptake value in positron emission tomography/computed tomography (PET/CT). The Mayo model [8] that was developed in 1997 includes older age, smoking history, cancer history, nodule diameter, location of nodule (especially the upper lobe), and spiculation. The VA model [9] developed in 2007 includes patient age, smoking history, and nodule diameter. The Brock model [10] developed in 2013 includes age, family history of lung cancer, sex, nodule size, emphysema, nodule count, location of the nodule in the upper lobe, spiculation and part-solid nodule. The Herder model was developed in 2005 at a single center, by analyzing data from 106 patients who underwent ^18^FDG-PET, to allow optimization of the prior Mayo model [11] and has already been reported to be a more useful model for predicting malignancy versus the other models [7,12,13].

However, because ^18^FDG is a marker of glucose metabolism and is not a specific tracer for malignancy, inflammatory lung lesions, such as tuberculous granuloma and parasite infection, can mimic malignancy and yield false-positive results on PET/CT scans. Moreover, given that the prevalence of tuberculosis among the Korean population in endemic areas is in the intermediate range (70–90/100,000 persons/year) [14], various malignancy prediction models for pulmonary nodules should be considered in Korean adults. Therefore, in this study, we aimed to compare the predictive power of these four models in patients with biopsy-proven pulmonary lung nodules at a single center in Korea.

## Materials and methods

### Study population

**Fig 1:**
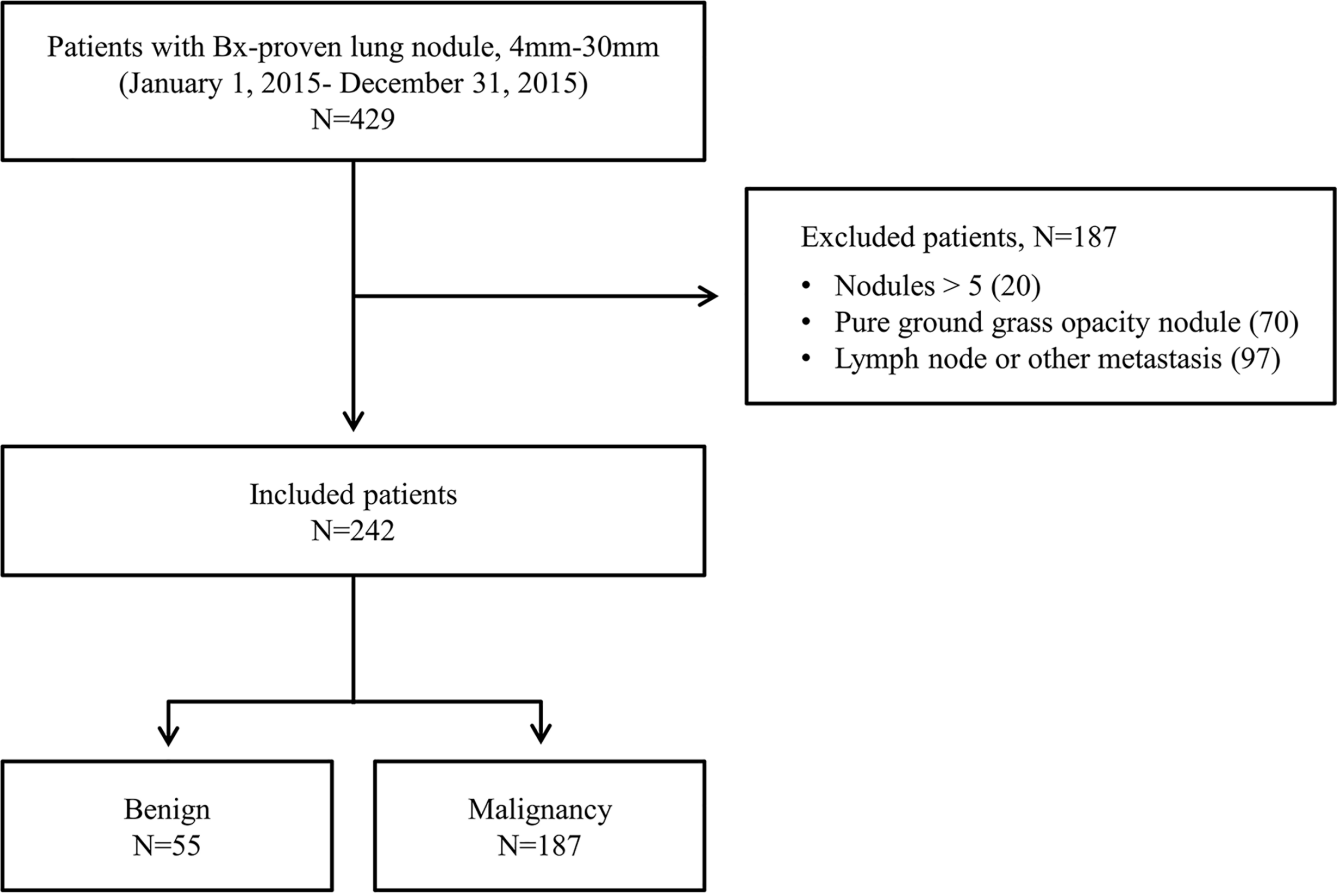
Study patients.

We retrospectively reviewed the medical records of 429 consecutive adult patients, with pulmonary nodules 4–30 mm in size, who underwent histopathologic confirmation at the Samsung Medical Center (a 1,979-bed referral hospital in Seoul, South Korea) between January 1, 2015 and December 31, 2015. Of these patients, 20 with more than five nodules, 70 with pure ground glass nodules, and 97 with lymphadenopathy or suspected metastatic disease on chest CT were excluded. As a result, 242 patients were included in the study, and 242 nodules that were confirmed by surgical resection or percutaneous needle aspiration were analyzed; of these, 187 (77.2%) were malignant, and the remaining 55 (22.8%) were benign (Fig 1). PET/CT was performed in 227 patients (93.8%).

The Institutional Review Board of Samsung Medical Center approved this study and permitted the review and publication of patient records (IRB No.2017-04-002). The requirement for informed consent of individual patients was waived given the retrospective nature of the study.

### Model for predicting malignancy

In this study, the probability of malignancy of pulmonary nodules was calculated using four models (Mayo [8], VA [9], Brock [10], and Herder [2]). Three models (Mayo [8], VA [9], and Brock [10]) are based on clinical values and CT findings. The fourth model (Herder [2]) adds characteristics of PET/CT in the Mayo Clinic [8] models. In accordance with Al-Ameri et al. [7], the nodules were classified according to their ^18^FDG avidity (standardized uptake value [SUV] or maximum standardized uptake value [SUV_max_]), as follows: faint uptake, SUV_max_ ≤ 2.5; moderate uptake, SUV_max_ 2.6–10.0; and intense uptake, SUV_max_ > 10.0 [7].

### Statistical analysis

The data are reported as numbers (%) for categorical variables and as medians (interquartile range, IQR) for continuous variables. We compared categorical variables using chi-squared test or Fisher’s exact test, and continuous variables using the Mann–Whitney *U* test. All tests were two-sided, and a P-value < 0.05 was deemed to indicate statistical significance. A receiver operating characteristic (ROC) curve was constructed, and the area under the ROC curve (AUC) was calculated. To compare the AUC values between two models, the nonparametric approach of DeLong et al. [15] was used, and Bonferroni correction was applied to adjust for multiple comparisons. All statistical analyses were performed using SPSS (ver. 23.0; IBM Corp., Armonk, NY, USA) and R software (ver. 3.0.3; R Development Core Team, Vienna, Austria).

## Results

### Baseline characteristics of the study patients and nodules

The baseline characteristics of the study patients and nodules are summarized in Table 1. The median age of the 242 patients was 61.0 years (IQR: 54.0–67.0 years); 112 (46%) patients were male and 148 (61%) patients were never smokers. The mean number of nodules was 1.4 for benign nodules and 1.5 for malignant nodules.

**Table 1:** Baseline characteristics of the study patients and nodules.

Clinical characteristics	Total (N = 242, 100%)^a^	Benign (n = 55, 23%)	Malignant (n = 187, 77%)	p-value
Age, years	61.0 (54.0–67.0)	57.0 (50.3–65.0)	62.0 (55.0–68.0)	0.316
Sex, male	112 (46)	23 (42)	89 (48)	0.746
Never smoker	148 (61)	39 (71)	109 (58)	0.521
Comorbidities				
Diabetes mellitus	34 (14)	11 (20)	23 (12)	0.138
Hypertension	59 (24)	15 (27)	44 (24)	0.601
Emphysema	21 (9)	2 (4)	19 (10)	0.261
Tuberculosis history	22 (9)	6 (11)	16 (9)	0.791
Idiopathic pulmonary fibrosis	4 (2)	1 (2)	3 (2)	0.999
Number of nodule	1.5	1.4	1.5	0.164
Nodule characteristics				
Nodule size	20.0 (15.0–25.0)	19.0 (15.0–28.0)	20.0 (15.0–24.0)	0.702
Part solid nodule	121 (50)	12 (22)	109 (58)	< 0.001
Spiculation	32 (13)	2 (4)	30 (16)	0.024
SUV_max_^b^	2.6 (1.4–4.4)	3.1 (1.6–3.7)	2.6 (1.4–4.8)	0.165
Faint	114 (46)	24 (44)	90 (48)	
Moderate	93 (38)	22 (40)	71 (38)	
Intense	20 (8)	2 (4)	18 (10)	
Previous extra-thoracic cancer	19 (8)	4 (7)	15 (8)	0.999
Family history of lung cancer	34 (14)	5 (10)	29 (16)	0.275

SUV, standardized uptake value.

^a^The data are presented as numbers (%) or medians (interquartile range).

^b^Fifteen patients, including seven patients in the benign nodule group and eight in the malignant group, lacked SUV_max_ values because they did not undergo PET/CT.

The mean size of the 242 nodules was 20.0 mm (IQR: 15.0–25.0 mm); 121 (50%) had part-solid nodules, 32 (13%) showed characteristics of spiculation, and the mean SUV was 2.6 (IQR: 1.4–4.4). In total, 114 (46%) nodules showed faint uptake, 93 (38%) showed moderate uptake, and 20 (8%) showed intense uptake. Of the 55 benign nodules, 24 (44%) showed faint uptake, 22 (40%) showed moderate uptake, and 2 (4%) showed intense uptake. In addition, of the 187 malignant nodules, 90 (48%) showed faint uptake, 71 (38%) showed moderate uptake, and 18 (10%) showed intense uptake. There was no significant difference in SUV_max_ between the two nodule groups. Nineteen (19%) patients had a history of extra-thoracic cancer diagnosed within the last 5 years, and thirty-four patients (14%) had a family history of lung cancer.

### Receiver operating characteristic (ROC) curves for the four risk prediction models

**Fig 2:**
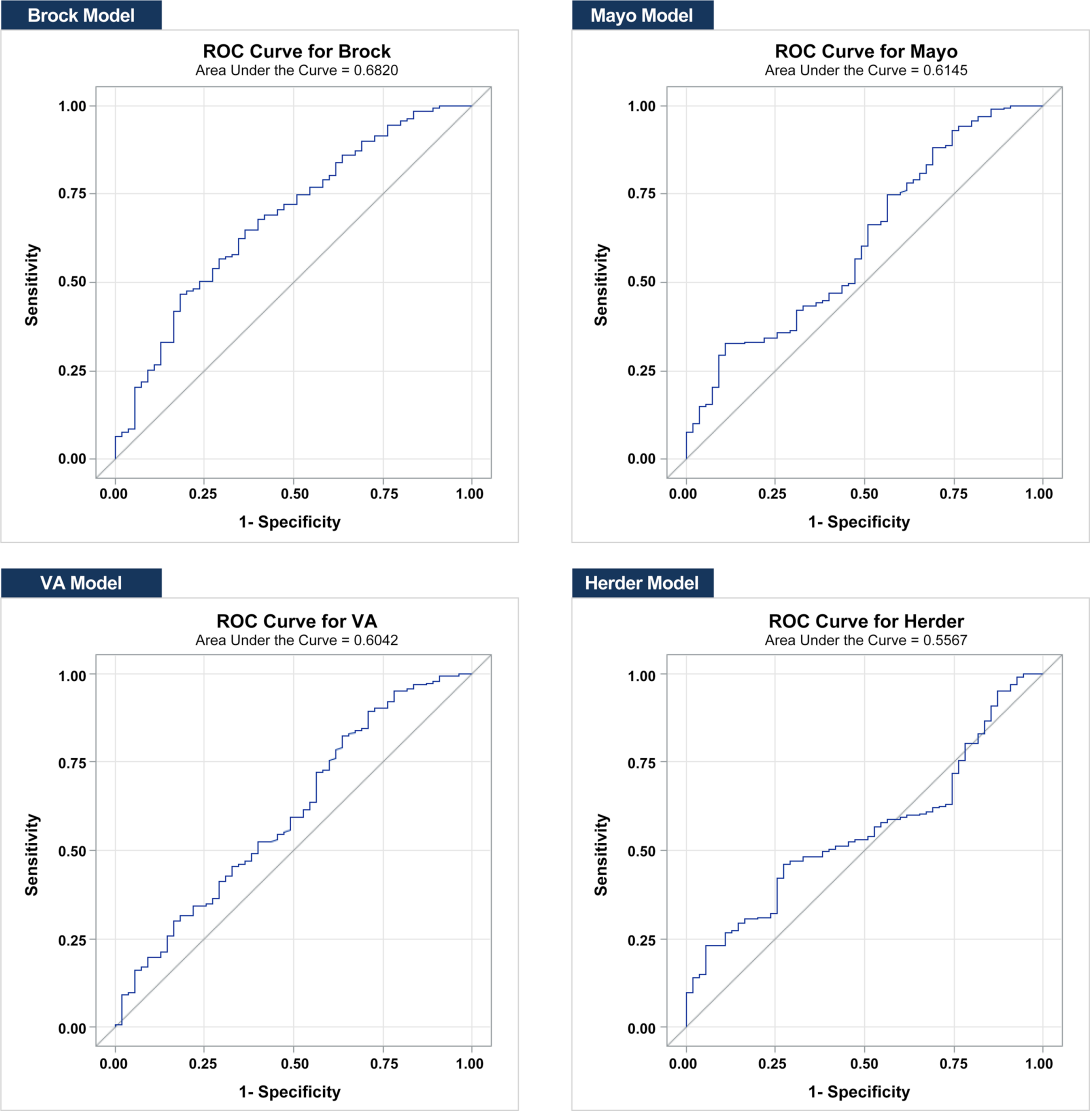
Receiver operator characteristic curves for the four risk prediction models.

**Fig 3:**
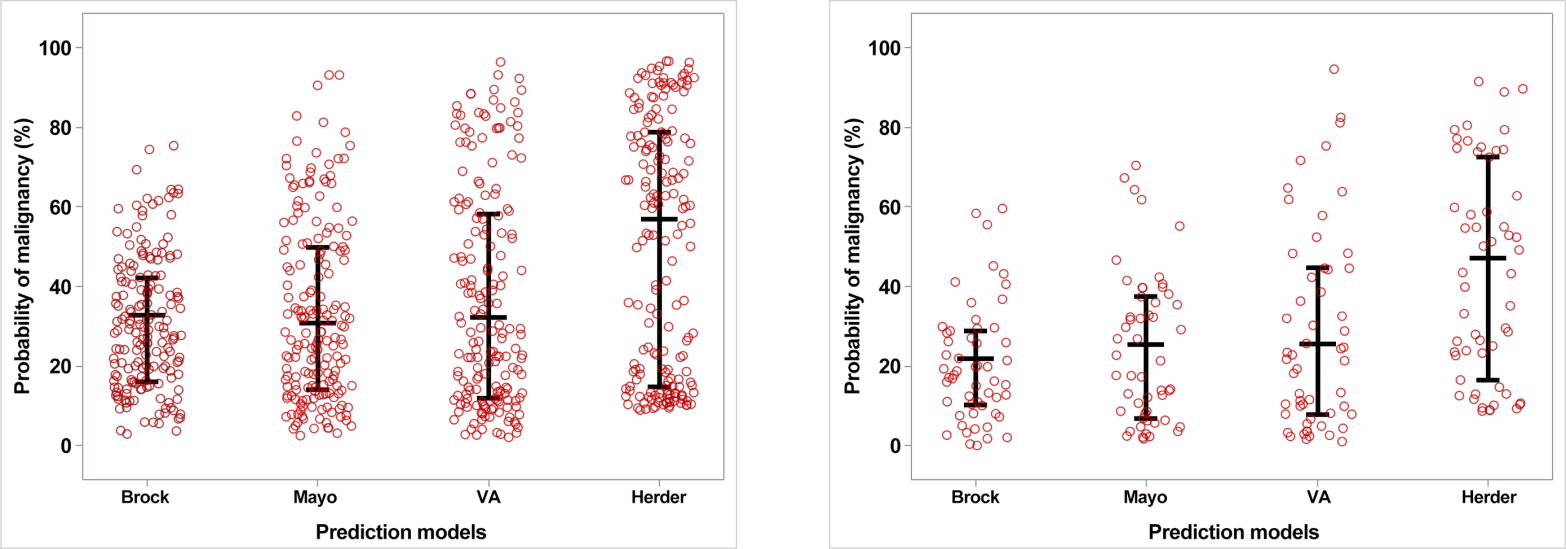
Distribution of the probability of malignancy according to the four risk prediction models.

The area under the ROC curve values (AUC, 95% CI) for each model were as follows (Fig 2): Mayo, 0.615 (0.528–0.701); VA, 0.604 (0.516–0.692); Brock, 0.682 (0.601–0.763); Herder, 0.557 (0.476–0.637). The predictive power of the Brock model was higher than that of the other models, and the Herder model had the lowest predictive power. The performance of the Herder model (AUC: 0.5567) was not statistically significantly different from that of the Mayo model (vs. Herder, p = 0.576) or the VA model (vs. Herder, p = 0.999), and there were no differences among the three models in the ability to determine the probability of malignancy in pulmonary nodules. However, compared with the Brock model, the Herder model showed a significantly lower ability to predict malignancy (adjusted p = 0.0132). The distribution of the probability of malignancy according to the four risk prediction models is shown in Fig 3.

### Decision analysis for the four risk prediction models

**Fig 4:**
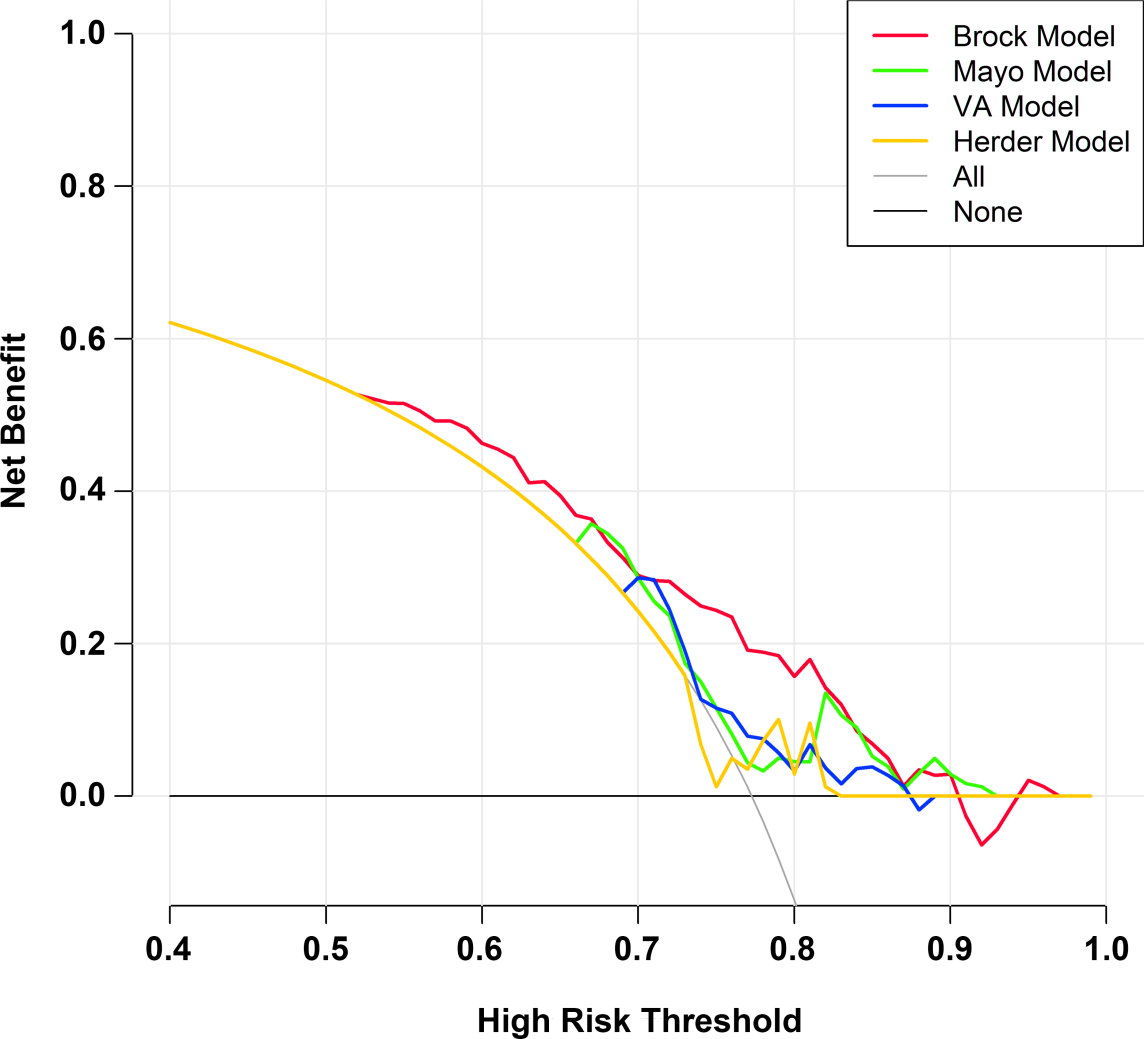
Decision curve analysis for the four risk prediction models.

We also evaluated the malignancy probability thresholds informing clinical decision-making of the American College of Chest Physicians (ACCP) [16] and the British Thoracic Society (BTS) [17] (Table 2): ACCP guidelines, observe (< 5%), undetermined (5–65%), surgery (> 65%); and BTS guidelines, observe (< 10%), undetermined (10–70%), surgery (>70%). As shown in Table 2, the false-positive rate was highest with the Herder model (up to 6%) using both the ACCP and BTS thresholds, while the true negative rate with the Brock model was up to 4–5% using the ACCP and BTS thresholds. The decision curves for the four models also showed that the Brock model had the highest power for discriminating malignant nodules, while the Herder model showed the lowest discriminatory power (Fig 4).

**Table 2:** Decision analysis using the ACCP and BTS thresholds in 242 confirmed nodules.

	Brock	Mayo	VA	Herder
ACCP				
T.P	3 (1)	27 (11)	36 (15)	74 (30)
T.N	9 (4)	10 (4)	11 (5)	0 (0)
F.P	0 (0)	2 (1)	5 (2)	14 (6)
F.N	3 (1)	8 (3)	9 (4)	0
Undetermined	227 (94)	195 (81)	181 (74)	154 (64)
BTS				
T.P	2 (1)	14 (6)	33 (14)	64 (26)
T.N	13 (5)	17 (7)	17 (7)	5 (2)
F.P	0 (0)	1 (1)	5 (2)	14 (6)
F.N	16 (7)	29 (12)	30 (12)	9 (4)
Undetermined	211 (87)	181 (74)	157 (65)	150 (62)

The data are presented as numbers (%). ACCP, American College of Chest Physicians; BTS, British Thoracic Society; VA, Veterans Association; T.P, true positive; T.N, true negative; F.P, false-positive; F.N, false-negative.

### Histopathological results of nodules

Histopathological results of the pulmonary nodules of the study patients are shown in Table 3.Of the 55 confirmed benign nodules, granuloma was the most common type (n = 28, 51%), followed by fibrin (n = 10, 18%), organizing pneumonia (n = 6, 11%), hamartoma (n = 4, 7%), carcinoid (n = 3, 5%), and others (n = 4, 7%). Of the 28 granuloma nodules, 23 (82%) were tuberculosis granuloma, 3 (11%) were actinomycosis granuloma, and 2 (7%) were cryptococcosis granuloma. Of the 187 malignant nodules, 172 (92%) were adenocarcinoma, and the remaining 15 (8%) were squamous cell carcinoma.

**Table 3:** Histopathological results of pulmonary nodules.

Biopsy results	Number (%)
Benign	(n = 55, 100%)
Granuloma	28 (51)
Tuberculosis	23/28
Actinomycosis	3/28
Cryptococcosis	2/28
Fibrin	10 (18)
Organizing pneumonia	6 (11)
Hamartoma	4 (7)
Carcinoid	3 (5)
Others^a^	4 (7)
Malignancy	(n = 187, 100%)
Adenocarcinoma	172 (92)
Squamous cell carcinoma	15 (8)

^a^Mucinous nodule, calcification nodule, lymphocyte aggravation, and sclerosing pneumocytoma were classified into the “Others” category.

## Discussion

Malignancy prediction models for pulmonary nodules should be applied with due consideration afforded to characteristics that may vary according to geographic region. The majority of malignancy prediction models were developed with Western populations in mind, and as such they are limited in their applicability to Asian populations. No universally applicable models for determining malignancy in pulmonary nodules are available, and the development of any such model should be executed with carefully and compared against the various extant risk models.

To our knowledge, this is the first study comparing the four models used to predict the probability of malignancy in the Korean population, especially in a tuberculosis-endemic area, to show how the various prediction models could be applied to local populations. Our results indicated that the Herder prediction model including the ^18^FDG uptake value was not better than the other models in predicting malignant nodules, suggesting the limited utility of considering PET/CT in the malignancy prediction process in populations within endemic areas for benign inflammatory nodules, such as tuberculosis.

One explanation for our results is that PET/CT resulted in false-positive findings for benign conditions, including infection, inflammation (soft tissue trauma, collagen diseases), and granulomatous infections (sarcoidosis, tuberculosis) [18–21]. In our study, there was no difference in SUV_max_ on PET/CT between benign and malignant nodules. Moreover, half (24/48) of the benign nodules showed moderate or intense SUV values on PET/CT. Thus, before assessing malignancy risk using a prediction model, physicians should consider whether the prediction models are useful for their patient populations, because there is geographical variation in the prevalence of granulomatous disease [7]. Therefore, physicians should also be aware of the possibility of false-positive findings when applying malignancy prediction models including PET/CT findings.

The differences and similarities of the four prediction models are as follows. In all models, the risk of malignancy increased with age and nodule size. In the Mayo and VA models [8–9], smoking history is included as an indicator to predict the malignancy of pulmonary nodules; in particular, the VA model includes the time of stopping smoking as a predictor [21]. The Mayo and Brock models [8,10] include the location of nodules and spiculation as predictors of malignancy, but the VA [9] model does not. In predicting the malignancy of pulmonary nodules, the VA and Brock models [9–10] exclude cancer history, whereas the Mayo and Herder models [8,11] include extrathoracic cancer more than 5 years prior. The Herder model is based on Mayo models including PET-CT characteristics [11]. Recent studies have reported that the Herder model incorporating FDG avidity has the highest accuracy in predicting the malignancy of pulmonary nodules, but this remains a subject of debate [2,7].

In our study, the AUCs for all models was lower than those in previous studies. For example, although the AUC of the Brock model in our study (0.682; 95% CI: 0.6009–0.7630) was higher than that of the other models, it was lower than that in the original article by McWilliams et al. (AUC: 0.96; 95% CI: 0.93–0.98) [10]. In addition, the AUC of the other models was lower than that in the original article. For example, the AUC of the Mayo model in our study (AUC: 0.6145; 95% CI: 0.5283–0.7008) was lower than that reported in the original article by Swensen et al. (AUC: 0.833; 95% CI: 0.811–0.855) [8], and the AUC of the VA model (AUC: 0.6042; 95% CI: 0.5162–0.6922) was also lower than that reported in the original article by Michael et al. (AUC: 0.78; 95% CI: 0.73–0.83) [9]. The Herder score including the patients who underwent PET-CT had the lowest accuracy in predicting the malignancy of pulmonary nodules in our patients (AUC: 0.5567; 95% CI: 0.4763–0.6371), and showed a worse performance than that seen in the original report (AUC: 0.92; 95% CI: 0.87–0.97) [11]. Several factors could explain this result. First, the lower AUC values in our study might be due to differences in the methods used to enroll the patients. In the present study, patients with pulmonary nodules confirmed by biopsy were retrospectively identified, a strategy that was different from that used in other studies. Second, compared with western populations, the incidence of lung cancer is higher in non-smoking, middle-aged Asian women. Third, our results were determined according to the prevalence of different types of benign nodule. In our study, 77% of lesions were malignant nodules, and 33% were benign nodules; of the benign nodules, granulomas accounted for 51% of the cases and showed high uptake in PET-CT.

Our study population may reflect a degree of selection bias: as our hospital is a tertiary referral center and most patients were referred with suspected malignant nodules, the rate of malignancy among our study population was relatively higher than has been seen in previous studies. Moreover, we retrospectively analyzed patients with biopsy-proven nodules for whom surgery was strongly recommended, and therefore some patients whose nodules had not yet been surgically confirmed may have been excluded. Additionally, patients presenting with very small nodules may have undergone observation without further diagnostic evaluation, such as PET-CT. All of these factors may have had a bearing on our results.

As we also included nodules that had been confirmed by percutaneous needle aspiration, and not only by surgical resection, the median nodule size and incidence of malignancy were relatively high, possibly contributing further to selection bias. Finally, we used the categorized, semiquantitative SUV_max_ values applied by Al-Ameri et al., which differs from the approach of Herder et al. [7,11].

In conclusion, we retrospectively evaluated four models for predicting malignancy in patients with biopsy-proven lung nodules. The highest AUC value was seen for the Brock model, but there was no significant difference between this value and those of the Mayo model and VA models. However, the Brock model showed significantly higher accuracy for predicting malignancy than the Herder model, which included the ^18^FDG uptake value, indicating the limited utility of PET/CT for predicting malignancy. When using prediction models to screen for the risk of malignancy of pulmonary nodules, physicians should consider the effects of regional differences, for example in terms of the prevalence of granulomatous disease.

## Supporting information

S1 Dataset (XLSX)

## References

[1] Jacobson FL. Multidetector-row CT of lung cancer screening. Semin Roentgenol. 2003; 38:168–75. 12854440 10.1016/s0037-198x(03)00019-1

[2] Perandini S, Soardi GA, Larici AR, Del Ciello A, Rizzardi G, Solazzo A, et al Multicenter external validation of two malignancy risk prediction models in patients undergoing 18F-FDG-PET for solitary pulmonary nodule evaluation. Eur Radiol. 2017; 27:2042–6. 10.1007/s00330-016-4580-3 27631108

[3] Aberle DR, Adams AM, Berg CD, Black WC, Clapp JD, Fagerstrom RM, et al Reduced lung-cancer mortality with low-dose computed tomographic screening. N Engl J Med. 2011; 365:395–409. 10.1056/NEJMoa1102873 21714641 PMC4356534

[4] Croswell JM, Baker SG, Marcus PM, Clapp JD, Kramer BS. Cumulative incidence of false-positive test results in lung cancer screening: a randomized trial. Ann Intern Med. 2010; 152:505–12, w176-80. 10.7326/0003-4819-152-8-201004200-00007 20404381

[5] Bach PB, Mirkin JN, Oliver TK, Azzoli CG, Berry DA, Brawley OW, et al Benefits and harms of CT screening for lung cancer: a systematic review. Jama. 2012; 307:2418–29. 10.1001/jama.2012.5521 22610500 PMC3709596

[6] Wood DE, Eapen GA, Ettinger DS, Hou L, Jackman D, Kazerooni E, et al Lung cancer screening. J Natl Compr Canc Netw. 2012; 10:240–65. 22308518 10.6004/jnccn.2012.0022 PMC6467530

[7] Al-Ameri A, Malhotra P, Thygesen H, Plant PK, Vaidyanathan S, Karthik S, et al Risk of malignancy in pulmonary nodules: A validation study of four prediction models. Lung Cancer. 2015; 89:27–30. 10.1016/j.lungcan.2015.03.018 25864782

[8] Swensen SJ, Silverstein MD, Ilstrup DM, Schleck CD, Edell ES. The probability of malignancy in solitary pulmonary nodules. Application to small radiologically indeterminate nodules. Arch Intern Med. 1997; 157:849–55. 9129544

[9] Gould MK, Ananth L, Barnett PG. A clinical model to estimate the pretest probability of lung cancer in patients with solitary pulmonary nodules. Chest. 2007; 131:383–8. 10.1378/chest.06-1261 17296637 PMC3008547

[10] McWilliams A, Tammemagi MC, Mayo JR, Roberts H, Liu G, Soghrati K, et al Probability of cancer in pulmonary nodules detected on first screening CT. N Engl J Med. 2013; 369:910–9. 10.1056/NEJMoa1214726 24004118 PMC3951177

[11] Herder GJ, van Tinteren H, Golding RP, Kostense PJ, Comans EF, Smit EF, et al Clinical prediction model to characterize pulmonary nodules: validation and added value of 18F-fluorodeoxyglucose positron emission tomography. Chest. 2005; 128:2490–6. 10.1378/chest.128.4.2490 16236914

[12] Perandini S, Soardi GA, Motton M, Dallaserra C, Montemezzi S. Limited value of logistic regression analysis in solid solitary pulmonary nodules characterization: a single-center experience on 288 consecutive cases. J Surg Oncol. 2014; 110:883–7. 10.1002/jso.23730 25088475

[13] Isbell JM, Deppen S, Putnam JB Jr., Nesbitt JC, Lambright ES, Dawes A, et al Existing general population models inaccurately predict lung cancer risk in patients referred for surgical evaluation. Ann Thorac Surg. 2011; 91:227–33; discussion 33. 10.1016/j.athoracsur.2010.08.054 21172518 PMC3748597

[14] World Health Organization. Global tuberculosis report. http://www.who.int/tb/publications/global_report/en/ (accessed 17.04.01). 2016

[15] DeLong ER, DeLong DM, Clarke-Pearson DL. Comparing the areas under two or more correlated receiver operating characteristic curves: a nonparametric approach. Biometrics. 1988; 44:837–45. 3203132

[16] Gould MK, Donington J, Lynch WR, Mazzone PJ, Midthun DE, Naidich DP, et al Evaluation of individuals with pulmonary nodules: when is it lung cancer? Diagnosis and management of lung cancer, 3rd ed: American College of Chest Physicians evidence-based clinical practice guidelines. Chest. 2013; 143:e93S–e120S. 10.1378/chest.12-2351 23649456 PMC3749714

[17] Callister ME, Baldwin DR, Akram AR, Barnard S, Cane P, Draffan J, et al British Thoracic Society guidelines for the investigation and management of pulmonary nodules. Thorax. 2015; 70 Suppl 2:ii1–ii54. 10.1136/thoraxjnl-2015-207168 26082159

[18] Patz EF Jr., Lowe VJ, Hoffman JM, Paine SS, Burrowes P, Coleman RE, et al Focal pulmonary abnormalities: evaluation with F-18 fluorodeoxyglucose PET scanning. Radiology. 1993; 188:487–90. 10.1148/radiology.188.2.8327702 8327702

[19] Knight SB, Delbeke D, Stewart JR, Sandler MP. Evaluation of pulmonary lesions with FDG-PET. Comparison of findings in patients with and without a history of prior malignancy. Chest. 1996; 109:982–8. 8635381 10.1378/chest.109.4.982

[20] Gupta NC, Maloof J, Gunel E. Probability of malignancy in solitary pulmonary nodules using fluorine-18-FDG and PET. J Nucl Med. 1996; 37:943–8. 8683316

[21] Liu BJ, Dong JC, Xu CQ, Zuo CT, Le JJ, Guan YH, et al Accuracy of 18F-FDG PET/CT for lymph node staging in non-small-cell lung cancers. Chin Med J (Engl). 2009; 122:1749–54. 19781319

